# Assessment of Hydration Status and Blood Pressure in a Tertiary Care Hospital at Al-Khobar

**DOI:** 10.7759/cureus.27706

**Published:** 2022-08-05

**Authors:** Ahmed S Mohammedin, Abir H AlSaid, Abdulaziz M Almalki, Abdulkarim R Alsaiari, Fahad N Alghamdi, Alwaleed A Jalalah, Abdulaziz F Alghamdi, Noor-Ahmed Jatoi

**Affiliations:** 1 Internal Medicine, King Fahd University Hospital, Imam Abdulrahman Bin Faisal University Hospital, Al-Khobar, SAU; 2 Geriatric Medicine Department, Faculty of Medicine, Ain Shams University, Cairo, EGY; 3 Internal Medicine (Pulmonology), King Fahd University Hospital, Imam Abdulrahman Bin Faisal University Hospital, Al-Khobar, SAU; 4 Internal Medicine, College of Medicine, Imam Abdulrahman Bin Faisal University Hospital, Al-Khobar, SAU; 5 Internal Medicine (Vascular Medicine), King Fahd University Hospital, Imam Abdulrahman Bin Faisal University Hospital, Al-Khobar, SAU

**Keywords:** hydration status, total body water, extracellular water, intracellular water, bioelectrical impedance, hemodynamic measurements, blood pressure, hypertension

## Abstract

Background

High blood pressure is a major cardiovascular risk factor. It is a leading cause of increasing morbidity and mortality worldwide. One-third of the adult population worldwide suffers from hypertension. Salt intake, obesity, decreased physical activity, and smoking are well known to increase blood pressure. Fluid retention is the main contributing factor to primary hypertension and adversely affects the cardiovascular system. The emerging evidence suggests a relationship between blood pressure and hydration status. Our study aims to assess the correlation between hydration status and blood pressure. We aim to assess the hydration status in subjects with normal and high blood pressure and to investigate the association of hydration status with hemodynamic measurement.

Methodology

This cross-sectional and observational study included adult (>18 years) male and female subjects who agreed to participate. In total, 235 subjects were recruited by convenience sampling from (1) patients and caregivers attending geriatric and internal medicine clinics, and (2) visitors coming to King Fahad University Hospital at Al-Khobar. There were five patients on oral diuretics who were excluded from the study. Data were collected from September 2021 to March 2022. Hydration status was measured by a bioelectrical impedance analyzer (Bioscan 920, Maltron International Ltd. Rayleigh, UK). Hemodynamic measurements included heart rate per minute, systolic blood pressure, diastolic blood pressure, pulse pressure (the difference between systolic and diastolic blood pressure), and mean arterial pressure calculated as blood diastolic pressure plus one-third of pulse pressure. Statistical analyses were performed using SPSS statistics for windows, version 28.0 (IBM Corp., Armonk, NY, USA). Descriptive data were reported using means with standard deviations for numerical data and relative frequencies (percentage) for categorical data. P-values of less than 0.05 were considered statistically significant. Comparison between groups was done using a one-way analysis of variance test.

Results

Extracellular water percentage was higher in hypertensive (45.0 ± 2) than prehypertensive (43.5 ± 3) or normotensive (43.0 ± 2) (p = 0.001) subjects. In contrast, intracellular water percentage and total body water percentage were significantly negatively related to hypertension status.

Conclusions

Our results have shown a strong association between hypertension status and hydration parameters. In our study, hypertensive subjects tended to have lower total body water percentage and intracellular water percentage (bioimpedance value) than normotensive subjects. This might promote more research regarding the relationship between hypohydration and cardiovascular disease pathophysiology. This outcome should raise awareness about proper hydration as hypohydration can be a causative factor for hypertension.

## Introduction

Hypertension is a major cardiovascular risk factor for morbidity and mortality worldwide [1]. Undiagnosed and untreated hypertension can lead to significant complications, including heart failure, stroke, nephropathy, and peripheral artery disease [1]. Epidemiological data by Mills et al. suggest that hypertension affected nearly one-third of the adult population worldwide in 2010 [2]. Locally, data collected between 1995 and 2000 showed that hypertension affected 26.1% of the adult Saudi population, and the prevalence was rising [1].

Water constitutes 63% of the adult body; it is distributed between the compartments of intracellular water (ICW, 67%) and extracellular water (ECW, 33%) [3]. The movement between the two compartments occurs by passive and active diffusion depending on the osmolarity of extracellular fluid (ECF), wherein an increase in the ECF osmolarity draws the water from the cells to outside the cells, causing ECW expansion at the expense of ICW [3]. The discrepancy in ECW/ICW is a predictive factor for all-cause mortality in malnourished and kidney dysfunction patients [4]. Although there are multiple techniques for the measurement of body fluid compartments, bioimpedance spectroscopy (BIS) remains a reliable, cost-effective technology that identifies body composition by sending electrical waves and estimating resistance values from very high to very low resistance numbers [5]. In addition, BIS allows measuring water compartments separately (total body water (TBW), ECW, and ICW) [5,6].

Cardiovascular parameters are highly affected by body fluid. A reduction in ECW causes blood pressure to drop [7]; this is mostly attributed to the decrease in the plasma volume [3,8]. However, ECW is more likely to be higher in those with high blood pressure [9]. In contrast with ECW, the relation between both TBW and ICW with blood pressure remains unclear. There are limited studies testing the change in ICW and TBW in patients with high blood pressure [9,10]. Our study aims to assess the relationship of hydration parameters in subjects with normal and high blood pressure to investigate the relationship between overhydration and underhydration with hemodynamic measurements in Al-Khobar, Saudi Arabia.

## Materials and methods

This prospective, cross-sectional, and observational study recruited adult (>18 years) male and female subjects. Subjects were recruited by convenience sampling from (1) patients and caregivers attending geriatric and internal medicine clinics, and (2) visitors coming to King Fahd University Hospital at Al-Khobar. We excluded people with severe heart failure, those on diuretics, clinically diagnosed kidney disease, severe dementia, terminal illness, pregnant women, and those who refused to consent. People with contraindications for using bioimpedance (e.g., pacemaker, amputated limb) were also excluded from the study. Data were collected from September 2021 to March 2022. Institutional Review Board (IRB) approval was obtained from the Imam Abdulrahman Bin Faisal University IRB office (IRB-UGS-2021-01-344).

Procedure and measurement

We utilized an interview-based questionnaire where the participants were informed about the study aims and procedure and then signed the informed consent. During the interview, investigators asked about demographic data including age and gender. History was taken from participants regarding cardiovascular risk factors and smoking status. Height, weight, and body mass index (BMI) were calculated and documented. Measurements of waist and hip circumferences were taken according to the World Health Organization protocol [11]. It indicates that waist measurement should be made at the level of the umbilicus, while hip measurement is taken around the widest portion of the buttocks using a non-stretchable tape measure positioned parallel to the floor, with the subjects positioned erect, arms to the sides, and feet approximated together. Subjects were advised to breathe out and not to tense their abdomen. All measurements were taken to the nearest centimeter [11]. The measurements were performed by a trained team, with full privacy ensured and gender-matched (males measuring males, and females measuring females).

Hemodynamic measurements

Procedures were explained and participants were instructed to lay supine and calm on the bed for five minutes before the examination. Blood pressure and heart rate were assessed from the left arm at the level of the left brachial artery using a digital sphygmomanometer (BP7200, Omron HealthCare. Inc., Kyoto, Japan) The mean of three blood pressure and heart rate readings were used for analysis, with a one-minute interval between each measurement. The difference between systolic blood pressure (SBP) and diastolic blood pressure (DBP) was used as the pulse pressure. The mean arterial blood pressure was calculated as DBP plus one-third of pulse pressure [12]. The blood pressure levels of participants were categorized according to the guidelines of the International Society of Hypertension [13] as (1) hypertensive with SBP ≥140mmHg and/or DBP ≥90 mmHg, and (2) prehypertensive with SBP 130-139 mmHg and/or DBP 85-89 mmHg [13].

Bioimpedance measurement

Hydration status was assessed by bioelectrical impedance using the Bioscan Model 920 (Maltron International Ltd., Rayleigh, UK). Bioscan model 920 is a non-invasive, painless, simple, inexpensive method that provides information about body composition, for example, fat mass, TBW, ECW, and ICW [5]. It was performed by applying four electrodes that were placed on two body aspects involving the wrist and ankle.

Statistical analysis

Data were analyzed by SPSS version 21 (IBM Corp., Armonk, NY, USA). Descriptive analysis was presented as percentage (%), mean, and standard deviation. Comparison between groups was done using the one-way analysis of variance test for variables with normal distribution, and a 95% confidence interval was applied.

## Results

The demographics and clinical characteristics of the study population are presented in Table 1. Of the 235 subjects, the mean age was 37.4 ± 17.3 years. Participants were mostly males, with an average height of 166.1 ± 8.2 cm, and an average weight of 76.4 ± 19 kg. Both overweight and obese participants constituted 59.2% of the study population. The mean waist/hip ratio (WHR) was within normal, and the mean fat mass percentage was 28 ± 9. Smokers comprised 32% of the study population. The means of hemodynamic measurements were within normal for SBP, DBP, pulse pressure, mean arterial pressure, and heart rate. BIS analysis showed that the TBW percentage was 52 ± 8, with an ICW percentage of 56 ± 2, and an ECW percentage of 43 ± 2.

**Table 1:** Demographic, clinical characteristics, and bioimpedance analysis data of study population. M = male; F = female; SD = standard deviation

Study variables	Mean ± SD
Age (years)	37.4 ± 17.3
Gender (M:F)	69:31
Height (cm)	166.1 ± 8.2
Weight (kg)	76.4 ± 19.1
Body mass index (kg/m^2^)	27.4 ± 6.2
Waist/Hip ratio	0.9 ± 0.1
Non-smoker:Smoker (%)	68:32
Hemodynamic measurements	
Heart rate (minute)	77 ± 13
Systolic blood pressure (mmHg)	133 ± 18
Diastolic blood pressure (mmHg)	82 ± 11
Pulse pressure (mmHg)	50 ± 11
Mean arterial pressure (mmHg)	99 ± 12
Normotensive (%)	56
Prehypertensive (%)	29
Hypertensive (%)	15
Bioimpedance analysis	
Total body water (L)	39 ± 7
Total body water (%)	52 ± 8
Extracellular water (L)	17 ± 3
Extracellular water (%)	43 ± 2
Intracellular water (L)	22 ± 4
Intracellular water (%)	56 ± 2
Fat mass (kg)	22 ± 12

Table 2 demonstrates that hypertension was significantly related to classic risk factors (age, gender, weight, body mass index, WHR ratio, and smoking). Comparison of body composition with blood pressure demonstrates that ECW (L) was higher in hypertensive (18.7 ± 4) than prehypertensive (17.1 ± 3) or normotensive subjects (16.6 ± 4) (p = 0.007). A similar relationship was found for ECW percentage as it was higher in hypertensive (45 ± 2) than prehypertensive (43.5 ± 3) or normotensive subjects (43 ± 2) (p = 0.001). Moreover, ICW percentage (p = 0.001) and TBW percentage (p = 0.001) were inversely related with hypertension status. Based on our results, fat mass (kg) was higher in hypertensive (30.1 ± 12) than prehypertensive (24.1 ± 12) or normotensive (20.1 ± 12) subjects (p = 0.001). On the other hand, TBW (L) and ICW (L) showed no significant relationship with hypertension status.

**Table 2:** Comparison between hypertension status and all related patient variables. M = male; F = female; BMI = body mass index; ECW = extracellular water; ICW = intracellular water; TBW = total body water

		Hypertension status			
Variables	Normotensive (n = 131)		Prehypertensive (n = 69)	Hypertensive (n = 35)	P-value
Age (years)	31 ± 14		41 ± 17	57 ± 12	0.001*
M:F	57:43		80:20	97:3	0.001*
Weight (kg)	72.7 ± 18.9		78.2 ± 18.2	86.2 ± 18.1	0.001*
BMI (kg/m^2^)	26.1 ± 5.9		27.6 ± 5.6	31.6 ± 6.4	0.001*
Waist/Hip ratio	0.8 ± 0.1		0.9 ± 0.1	1.0 ± 0.1	0.001*
Non-smoker:Smoker	77:23		52:48	37:63	0.001*
TBW (L)	38.3 ± 8		39.4 ± 6	40.9 ± 8	0.161
TBW (%)	54.6 ± 8		52.2 ± 9	48.4 ± 6	0.001*
ECW (L)	16.6 ± 4		17.1 ± 3	18.7 ± 4	0.007*
ECW (%)	43.0 ± 2		43.5 ± 3	45 ± 2	0.001*
ICW (L)	22.2 ± 5		22.5 ± 4	22.6 ± 4	0.857
ICW (%)	57.1 ± 2		56.8 ± 2	55 ± 2	0.001*
Fat mass (kg)	20.1 ± 12		24.1 ± 12	30.1 ± 12	0.001*

## Discussion

Our data demonstrated that TBW is lower in hypertensive patients. This may signify the role of chronic hypohydration on blood pressure pathophysiology. However, this decrease can be attributed to several factors, such as advancing age, increase in BMI, or use of diuretics in hypertensive participants (patients on diuretics were excluded from the start).

Total body water and blood pressure

Our data demonstrated that TBW percentage of body weight was lower in hypertensive patients. However numerous studies testing the acute effect of dehydration on blood pressure have reported contradicting results. Our study aligns with previous animal research that linked episodic dehydration with hypertension in animals with renal damage [14]. Another study found that decreased water intake was associated with higher blood pressure [15]. The exact mechanism remains unclear although several mechanisms could explain our findings. First, a possible cause is that chronic dehydration increases serum sodium which triggers some inflammatory mediators such as vascular cell adhesion molecule, endothelial-leukocyte adhesion molecule 1 (E-selectin), and monocyte chemoattractant protein 1 (MCP-1) [16]. Chronic inflammation can lead to endothelial layer dysfunction, which may impair peripheral artery vasodilatory capacity in healthy subjects [17]. Second, angiotensin II is thought to have a further role as angiotensin II infusion elicits endothelial dysfunction by stimulating reactive oxygen species [18]. Additionally, the vasoconstrictive effect of elevated angiotensin II in a hypohydration state can be another contributing factor [19]. Further studies suggest that chronic dehydration increases angiotensin II receptor number and density in blood vessels which further aggravates its vasoconstrictive effect [19]. The reviewed factors collectively contribute to the development of high blood pressure. However, our results might not be applicable to people with severe chronic kidney disease (CKD) as several studies have shown that an increase in body fluid increases the blood pressure in people with severe CKD [7,9]. Other factors are expected to contribute to the interaction between hydration and hypertension.

Extracellular water and blood pressure

We found a strong association between ECW and high blood pressure. This finding is in line with previous studies on peritoneal dialysis and CKD patients [7,9]. Several explanations have been proposed. The first explanation is that high ECW increases the venous return, which, in turn, increases cardiac output and vascular resistance. The second explanation is that people with high ECW have higher salt intake which is associated with high blood pressure [20]. The third explanation is that compensatory mechanisms for chronic dehydration may have a role such as abnormal activation of the renin-angiotensin-aldosterone system, Na/K pump dysfunction, and sympathetic nervous system dysregulation [21].

Intracellular water and blood pressure

Our results have demonstrated an inverse relationship between high blood pressure and ICW, which is consistent with some previous studies [21,22]. However, this relationship is unclear and needs further research. Kumari and Raghuvanshi have reported similar data to our results [22]. While a study by Cianci et al. reported a conflicting result [21]. Further investigation is needed to understand the exact mechanism by which ICW affects blood pressure.

Study limitations

There are several limitations to our study. First, the study sample was relatively small. Second, our sample was predominantly males as many females were unwilling to participate due to cultural aspects. Other limitations include the age difference between hypertensive and normal subjects, BMI difference, and no laboratory or clinical assessment to define dehydration status. Further research is needed to confirm our results and assess the interaction between hydration and high blood pressure.

## Conclusions

Our results have shown a strong association between hypertension status and hydration parameters. Hypertensive subjects tended to have lower TBW and ICW than normotensive subjects in our sample. This might promote more research regarding the relationship between hypohydration and cardiovascular disease pathophysiology. This outcome should raise awareness about proper hydration as hypohydration can be one of the risk factors of hypertension.
